# The Society of Information and the European Citizens’ Perception of Climate Change: Natural or Anthropological Causes

**DOI:** 10.1007/s00267-024-01961-x

**Published:** 2024-03-18

**Authors:** Fernando Mata, Maria Dos-Santos, Concha Cano-Díaz, Meirielly Jesus, Manuela Vaz-Velho

**Affiliations:** 1 CISAS—Center for Research in Agrifood Systems and Sustainability, Instituto Politécnico de Viana do Castelo, Viana do Castelo, Portugal; 2 Escola Superior de Comunicação Social, Instituto Politécnico de Lisboa, Lisboa, Portugal; 3 Dinâmia-CET—Centre for Socioeconomic and Territorial Studies, ISCTE—Centro Universitário de Lisboa, Lisboa, Portugal; 4 Escola Superior de Tecnologia e Gestão, Instituto Politécnico de Viana do Castelo, Viana do Castelo, Portugal

**Keywords:** Anthropogenic causes, Climate change, European Citizens, Natural causes, Perception

## Abstract

The scientific community has reached a consensus on humans’ important role as causative agents of climate change; however, branches of society are still sceptical about this. Climate change is a key issue for humanity and only the commitment to change human attitudes and lifestyles, at the global level, can be effective in its mitigation. With this purpose, it is important to convey the right message and prevent misinformation to manipulate people’s minds. The present study aims to understand the factors shaping European citizens’ thoughts on the causes of climate change. Using data from the European Social Survey 10 collected in 2022, we fitted statistical models using the people’s thoughts on causes of climate change (natural, anthropogenic or both) as dependent variables. As independent variables, we used the impact of the media through time spent on news and time spent on the internet, level of education, level of trust in scientists, awareness of online or mobile misinformation and gender. We concluded that the typical European citizen who believes in anthropogenic causes of climate change is a female, is more literate, trusts more in scientists, is younger, spends more time reading the news and has more awareness of misinformation presence in online and mobile communications.

## Introduction

### Anthropogenic and Natural Causes of Climate Change

Climate change (CC) is a complex phenomenon influenced by a variety of natural and anthropogenic factors. The understanding of the interplay between factors is crucial for assessing the current state of the climate and its change and is paramount to developing strategies to prevent anthropogenic CC and mitigate impacts. The Intergovernmental Panel on Climate Change (IPCC 2023) has pointed out several natural and anthropogenic causes of CC.

Human activities have significantly altered the concentrations of greenhouse gases in the atmosphere, contributing to CC. On the other hand, the destruction of forests not only increases the GGE but also reduces carbon sink activity where forests are major players (Nunes et al. 2020). Other anthropogenic activities contributing to CC include agriculture through activities such as livestock production (ruminants) and waste treatment processes in landfills releasing methane (Sundar et al. 2021).

Natural causes of CC include solar variability (Sundar et al. 2021) and the Milankovitch cycles influencing climate by affecting the distribution of sunlight but is a slow process taking tens of thousands of years to occur (Buis 2020).

With the rise of temperatures large amounts of previously trapped methane have also been released from the thawing permafrost (Knoblauch et al. 2018). The melting of ice caps in the polar regions has also a negative effect on global warming as these caps are also responsible for an albedo effect capable of reflecting 80% of the solar energy they receive back to space (Gschnaller 2020). The thawing permafrost and the ice cap melting are examples of a snowballing chain of events difficult to revert (Hansen et al. 2023).

### The Role of Media, Politics, Trustworthiness in Science and Education in Shaping People’s Perception of Climate Change

The perception of CC is a complex interplay of various factors that shape public understanding and attitudes. Media has been serving as a powerful mediator between scientific findings and public perception (Höttecke and Allchin 2020). The framing of CC narratives plays a crucial role in shaping individuals’ perceptions of this global challenge (Whitmarsh and Capstick 2018). Often, media coverage sensationalizes extreme weather events, emphasising the immediacy and severity of the issue. This focus on the dramatic can inadvertently lead to a biased perception of CC, emphasising natural causes over anthropogenic ones. Striking a balance in reporting, incorporating scientific consensus and highlighting solutions can contribute to a more nuanced understanding (Kock 2019).

CC has also become a political battleground, especially in the USA, with policy decisions influencing public discourse (O’Riordan and Jäger 2019; Busch and Judick 2021). Political ideologies can strongly shape the narrative surrounding CC, contributing to polarisation. In the USA, Republican political leaders have downplayed the human influence on CC to protect economic interests (Collomb 2018). The politicisation of CC can hinder collective efforts to address the issue by fostering scepticism and division among the public (Pepermans and Maeseele 2018).

Trustworthiness in science is fundamental to conveying information sustained on evidence, capable of creating awareness and correctly informing public perception of CC causes. Trust in scientists and scientific institutions can be influenced by factors such as perceived objectivity, transparency and the ability to communicate complex information effectively (Lacey et al. 2018). Scepticism fuelled by campaigns of misinformation, or the politicisation of science, can erode public trust (Farrell et al. 2019). Strengthening scientific communication, promoting transparency and countering misinformation are critical factors in fostering a public reliance on credible science to foster informed opinions on CC (Hornsey and Lewandowsky 2022).

Corporate interests and lobbying have a significant impact on CC narratives. Industries with a stake in maintaining the status quo may fund campaigns to introduce doubt on climate science or influence policymakers to resist regulatory measures (Grasso 2019). This can contribute to a narrative that emphasises natural factors over human-induced causes. Acknowledging and addressing the influence of lobbying in public discourse is crucial for conveying an informed and unbiased understanding of CC (McKie 2019).

Education is a key determinant in shaping long-term attitudes toward CC. The inclusion of comprehensive climate science education can empower individuals to critically evaluate information, fostering a scientifically literate society (Taimur and Sattar 2019). Efforts to enhance environmental education and promote critical thinking skills are essential in cultivating a population capable of understanding the complexities of the CC discourse (Shutaleva 2023).

### Framing People’s Perception of the Causes of Climate Change

The causes for CC have been debated since the middle of the last century (Bindoff et al. 2014; Stern and Kaufmann 2014), and mainly from 1988 when CC was first put on the policymakers’ agenda (Painter and Ashe 2012), which led to the UN Framework Convention on Climate Change (UN-FCCC) in 1992 (Leggett 2020). However, scepticism and denial of CC have also been accompanying scientific research on the matter (Anderegg et al. 2010). The debate between natural and anthropogenic causes of CC has also been subjected to discussion (Stern and Kaufmann 2014), but scientists believe in their experimental models to sustain the important role of human activity in CC (Anderegg et al. 2010; Bindoff et al. 2014; Stern and Kaufmann 2014).

Currently, it is established by the Intergovernmental Panel on Climate Change (IPCC) that CC is mainly driven by humans through increased emissions of gas with greenhouse effects (Parry et al. 2007). Nevertheless, scientists have also identified natural non-anthropogenic causes for CC (Hegerl et al. 2019; Stern and Kaufmann 2014). Limited predictive accuracy on CC comes from the scarcity of data before the 20th century which imposes limitations on long-term time series to model with accuracy long-term climate variations (Hegerl et al. 2019).

To mitigate the anthropogenic effects of CC, every person must be aware of the degree of importance of the problem so we can solve economic and societal changes (Mahalik et al. 2021). This is a global issue that can only be addressed through profound changes in attitudes and lifestyles, especially in developed countries. Therefore, an educated society capable of understanding what scientists communicate is desirable (Cordero et al. 2020). In that sense, educators have an important role to play and in fact, several works have been produced to understand how the CC message can be conveyed efficiently to pupils (Leal Filho et al. 2021; Ojala and Bengtsson 2019; Tolppanen et al. 2021) and students not only in formal education but also by society, parents and friends (Collado et al. 2019). Education has been recognized as a priority in assembling a global strategy to mitigate CC, since the UN-FCCC (Zhang and Ghorbani 2020).

An educated society is one capable of thriving in a society of information, to do so critical thinking needs to be developed to be able to identify false or misleading from honest information and to separate the wheat from the weed. Dubious information or ‘fake news’ has been growing on social media (Zhang and Ghorbani 2020) and are posing new challenges to society to make informed decisions and form informed opinions (Machete and Turpin 2020). The power of misinformation is so high that in 2016 after the EU referendum in the UK and the presidential elections in the USA, ‘post-truth’ was chosen as the word of the year by the Oxford Dictionary (Fernandez and Alani 2018). Several authors have claimed that misinformation has negatively impacted public opinion and policymakers in the adoption of the necessary CC mitigating measures (Cook et al. 2018; Van der Linden et al. 2017).

Political and industrial motivations have been pointed out as factors of CC denial (Farrell 2016; Hornsey et al. 2016), that justify inaction in mitigation efforts. The fossil fuel industry primarily has been accused of undermining scientific information (Supran and Oreskes 2017), which has been pointed out as a motivated denial of CC (Wong-Parodi and Feygina 2020b). Important politicians such as the USA ex-President Donald Trump are examples of how a single powerful individual can divide a large society, such as the American society. The American society went through a process of excessive denial and the divide was polarised by the two major political parties. Between the Republicans prevailed the CC denial, at least caused by human activity, while between the Democrats that was not the case (Dunlap et al. 2016). Nevertheless, in a study conducted by the Yale Program on Climate Change Communication, Americans were categorized into six different climate opinion audiences (alarmed, concerned, cautious, disengaged, doubtful and dismissive). The study revealed that 28% of Americans are alarmed, outnumbering the dismissive group (11%). Furthermore, the alarmed and concerned groups together constitute the majority of opinions, totaling 56%. Additionally, it was observed that the proportion of alarmed or concerned Republicans increased from 22% in 2022 to 28% in 2023 (Leiserowitz et al. 2023).

Public opinion in America about the causes of CC has been extensively dissected; however, the same cannot be said for Europe. The present study aims to understand European citizens’ thoughts on the causes of CC. With this aim established, we will research the impact of the media, the level of education, the level of trust in scientists, and the demography, in the perception of CC causes by European citizens. The research questions are: What is the European citizens’ position concerning CC causes? Natural processes, human activity, both natural processes and human activity? Do European citizens believe in CC? How do media consumption habits and critical thinking toward news affect European citizens’ perception of CC causes?

## Materials and Methods

### Data

Data are freely available and were retrieved from the European Social Survey (ESS) (ESS ERIC 2022a). It was collected between May 25 and September 18, 2022. The ESS is in its 10th edition and was conducted in 25 European countries. Data were collected using presential interviews mainly, however, because of the COVID-19 pandemic, some were done using web questionnaires or videoconferences.

The survey includes items related to the Europeans’ lives, including social indicators and conditions, attitudes and social behaviour, well-being and general health, attitudes and political behaviour and ideology, inclusivity of minorities and equality, inequality and social exclusion, national and cultural identity, media, values and religion, linguistics and language, family life and marriage (ESS ERIC 2022b).

The sampled universe includes persons aged over 15, with residence within private households, regardless of their language or legal status, citizenship, or nationality and took place in Austria, Bulgaria, Switzerland, Czechia, Germany, Estonia, Spain, Finland, France, Greece, Croatia, Hungary, Iceland, Italy, Lithuania, Montenegro, North Macedonia, Netherlands, Norway, Poland, Portugal, Serbia, Sweden, Slovenia and Slovakia. A total of 18,060 interviews were conducted.

### Variables Included in the Study

We aimed to understand what European citizens think about the causes of CC, we have selected, as a dependent variable (DV), the response to the question ‘Do you think that climate change is caused by natural processes, human activity or both?’. We have then selected several other variables of interest as independent variables (IVs) to explain the choices in the DV. The following were used as IVs:

Demographic: ‘Age’, ‘Gender’ and ‘Years of full-time education completed’.

Society of information: ‘Time spent watching, reading, or listening to news about politics and current affairs’, ‘How often do you use internet’, ‘How much online or mobile communications expose people to misinformation’ and ‘Trust in scientists’.

The DV was responded with categorical options (1—entirely by natural processes, 2—mainly by natural processes, 3—about equally by natural processes and human activity, 4—mainly by human activities and 5—entirely by human activities). The option ‘I don’t think climate change is happening’ was also available.

The IVs question about time spent with the news was answered in minutes per day. The IVs demographic questions had a choice of men or women for ‘Gender’ and years for ‘Age’ and ‘Years in full-time education completed’. The other IVs questions were answered on a 0–10 scale: from 0—not at all, to 10—completely, for the question about the ‘exposure of online and mobile communications to misinformation’; and from 0—no trust to 10— trust completely, for the question about trusting in scientists.

The question ‘How often do you use internet’ had categorical options to answer: 1—never, 2—only occasionally, 3—a few times a week, 4—most days and 5—every day. All the questions resulting in the DV, and IVs had as answering options ‘don’t know’, ‘refused to answer’, or ‘didn’t give an answer’ and the respective entries were omitted from the analysis.

### Statistical Procedure

The DV was rearranged in different formats to allow specific results. In the first model (‘extremes model’) we have analysed only the individuals responding to the extreme answers, therefore the individuals answering that CC is caused ‘entirely by natural causes’ or ‘entirely by human activity’. This dichotomy was fit to a binomial model with a logit link. In a second model (‘tendencies model’) we have joined together the individuals answering, CC is caused ‘entirely by natural causes’ with those answering, ‘mainly by natural processes’; the same was done with those answering, ‘entirely by human activity’ and ‘mainly by human activity’. Again a dichotomic DV was obtained to fit a binomial model with a logit link. The third model (‘full model’) considered the full range of five possible answers independently and without aggregation and therefore, a multinomial model with a logit link was fit to data.

Finally, to study the different motivations between individuals answering, ‘I don’t think climate change is happening’ and those acknowledging CC, a binomial model with a logit link was again chosen to fit data.

In all the models a backward stepwise selection of variables was implemented. The models were evaluated by the likelihood ratio chi-square test, and the variables in the model by the Wald chi-square test. The Akaike’s information criterion for the models is also given. Wald confidence intervals were produced for all the models’ parameters. The exponentiation of the parameters, giving the odds ratio for the variable was also calculated, as well as their Wald confidence intervals. All levels of significance were set to *p* < 0.05. The procedures were implemented via GENLIN routine for the binomial models and via CSLOGISTIC routine for the multinomial model, from the statistical package IBM Corp.® SPSS® Statistics, Armonk, NY, USA. Version: 28.0.1.1 (15). The odds ratio for the logistic regressions was also confirmed by Mann–Whitney *U* tests and the variables were checked for normal distribution where applicable, using the Kolmogorov–Smirnov test. The Spearman’s test was used for correlations between non-parametric variables.

## Results

### Descriptive Statistics

**Fig. 1:**
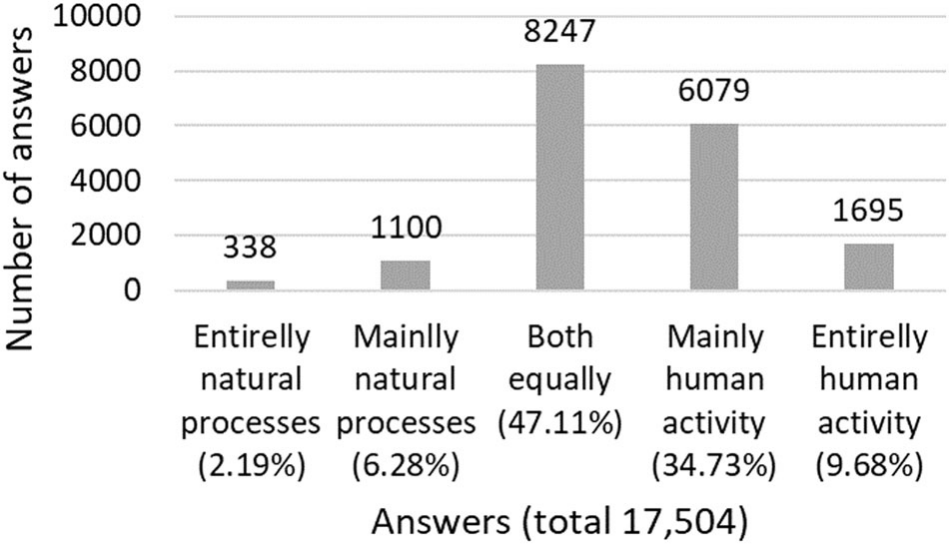
Graph of the distribution of answers. Distribution of answers to the question ‘Do you think that climate change is caused by natural processes, human activity or both?’

A total of 18,060 interviewees were sampled. Relatively to the IVs in this study, Table 1 reports the descriptive statistics. Relatively to the DV, 17,504 interviewees answered one of the five options of the question ‘Do you think that climate change is caused by natural processes, human activity, or both?’ and was considered in the main models analysed. The distribution of answers can be visualized in Fig. 1. Still, in relation to the DV, another 201 answered ‘I don’t think climate change is happening’ and this group was treated separately. Also, 355 interviewees were excluded from the analysis by refusing to answer (14), answering do not know (340) and the inexistence of answer (1).

**Table 1:** Descriptive statistics for the independent variables (covariates) included in the study

			Variables^a^				
			1	2	3	4	5
*N*	Valid		11,845	17,625	17,109	17,940	17,720
	Omitted		6215	435	951	120	340
Mean			6.79	78.56	6.78	50.89	13.11
Median			7	60	7	51	12.00
Standard error			2.460	0.850	0.019	0.138	0.020
Minimum			0	0	0	15	0
		25	5	30	10	36	11
Quartile		50	7	60	5	51	12
		75	9	90	7	66	15
Maximum			10	1200	9	90	40

^a^Variables 1 and 3 were responded on a 0–10 scale (from ‘not at all’ to ‘a great deal’). Variables: 1—‘Trust in scientists’, 2—‘Time (minutes per day) spent watching, reading or listening to news about politics and current affairs’, 3—‘How much online or mobile communications expose people to misinformation’, 4—‘Age’ (years), 5—‘Years of full-time education completed’ (years); Refusal to answer, not giving an answer, or answering ‘don’t know’ were omitted from the analysis

### The *Extremes Model*. Climate Change Caused Entirely by Natural Processes or Entirely by Human Activity

A model was successfully fitted to the subset of data considering the two most extreme answers only: CC is caused ‘entirely by natural processes’, and CC is caused ‘entirely by human activity’. The parameterisation is shown in Table 2.

**Table 2:** Logistic regression parameterisation of the *extremes model*

			95% Wald CI			95% Wald CI	
Parameter	*β*	Std error	lower	upper	Exp(*β*)	lower	upper
Trust in scientists**	−0.076	0.0244	−0.124	−0.029	0.926	0.883	0.972
Years in education***	−0.057	0.0149	−0.086	−0.027	0.945	0.918	0.973
Awareness of misinformation*	−0.051	0.0237	−0.098	−0.005	0.950	0.907	0.995

Likelihood ratio *χ*^2^ = 585, 3df, *p* < 0.001, AIC = 11,072; The model was adjusted without intercept. From the 18,060 individuals interviewed, 1264 (7.0% of the total) answered the extreme options: 227 answered ‘Climate change is entirely caused by natural processes’ and 1037 answered ‘Climate change is entirely caused by human activity’

‘Years in education’ —‘Years of full-time education completed’; ‘Awareness of misinformation’—‘How much online or mobile communications expose people to misinformation’; Climate change is caused ‘entirely by natural processes’ is modelled, climate change is caused ‘entirely by human activity’ is the reference

*p < 0.05; **p < 0.01; ***p < 0.001

The variables ‘Age’, ‘Gender’, ‘Time spent watching, reading or listening to news about politics and current affairs’ and ‘How often do you use the internet’ are not significant and did not enter the model. Using the odds ratio Exp(*β*) for the interpretation of the significant results:

- The probability for the answer CC is caused ‘entirely by natural processes’ conflicts with ‘Trust in scientists’. Per unit of change in ‘Trust in scientists’, there is a 7.4% (1 − 0.926 = 0.074) decrease in the probability of the answer being ‘entirely by natural processes’. Or the odds of answering CC is caused ‘entirely caused by human activity’ increase as ‘Trust in scientists’ increases. The result can also be confirmed by the significant differences (Mann–Whitney *Z* = −3.071, *p* < 0.01) found while testing how the two groups answering ‘entirely by human activity’ or ‘entirely by natural processes’ score ‘Trust in scientists’.
- The last significant variable in the model is ‘Years in full-time education completed’, with an odds ratio of 0.945 and therefore a decrease in the probability of the answer being ‘entirely by natural processes’ as time in education increases. Per unit of change in ‘Years in full-time education completed’ there is a 5.5% (1 − 0.945 = 0.055) decrease in the probability of the answer being ‘entirely by natural processes. The results are also confirmed by a Mann–Whitney *U* test (*Z* = −4.744, *p* < 0.001).
- The same tendency is observed when we consider ‘How much online or mobile communications expose people to misinformation’. Per unit of change in this variable, there is a 5.0% (1 − 0.950 = 0.05) decrease in the probability of the answer being ‘entirely by natural processes’. Again, the result is confirmed by the significant differences (Mann–Whitney *Z* = −4.603, *p* < 0.001) found while testing how the two groups rate ‘awareness of misinformation’, with ‘entirely by human activity’ rating higher than ‘entirely by natural processes.

### The *Tendencies Model*. Climate Change is Caused or Mainly Caused by Natural Processes or by Human Activity, but not Equally by Both

We have obtained a model of tendencies where the individuals giving more emphasis on one of the causes (natural or human) are considered. The parameterisation of the model is shown in Table 3. This model joins together the answers ‘mainly’ with ‘entirely’ both for ‘natural processes’ and for ‘human activity’; The answers ‘caused by both natural processes and human activity’ are ignored in this model.

**Table 3:** Logistic regression parameterisation of the *tendencies model*

			95% Wald CI			95% Wald CI	
Parameter	*β*	Std error	lower	upper	Exp(*β*)	lower	upper
Trust in scientists***	−0.106	0.0148	−0.135	−0.077	0.900	0.874	0.926
Years in education***	−0.067	0.0114	−0.089	−0.045	0.935	0.915	0.956
Awareness of misinformation***	−0.060	0.0147	−0.089	−0.031	0.942	0.915	0.969
Gender*** man	0.336	0.1836	−0.024	0.696	1.399	0.976	2.005
Women	0.038	0.1849	−0.325	0.400	1.038	0.723	1.492
News exposure*	0.001	0.0003	0.000	0.001	1.001	1.000	1.001

Likelihood ratio *χ*^2^ = 2509, 4df, *p* < 0.001, AIC = 3102; The model was adjusted without intercept as this is not significant (*p* > 0.05). Of the 18,060 individuals interviewed, 13,951 are excluded and 4109 are included in the model

‘Years in education’—‘Years of full-time education completed’; ‘Awareness of misinformation’—‘How much online or mobile communications expose people to misinformation’, 'News exposure’– ‘Time (minutes per day) spent watching, reading or listening to news about politics and current affairs’; ‘Climate change is entirely caused by natural processes’ is modelled, ‘Climate change is entirely caused by human activity’ is the reference (*β* = 0)

*p < 0.05; ***p < 0.001

The *Tendencies model* includes same variables as the *Extreme model* with similar response directions, however, includes other significant IVs (Gender and news exposure). Note the intensity of the variable effects (odds ratios) differs between models. The significant IVs in this model are the same as in the ‘extremes model’. In comparison, the direction of the results is also the same, but the intensity of the results is different for all the IVs. Therefore the odds ratios differ:

- The probability of the answer ‘natural processes’ decreases as ‘Trust in scientists’ increases. The probability decreases by 10% (1 − 0.9 = 0.1) per each score point more for ‘Trust in scientists. The result is confirmed by a Mann–Whitney *U* test (*Z* = −7.259, *p* < 0.001).
- The probability of the answer ‘natural processes’ decreases as ‘Years in education’ increases. The probability decreases by 6.5% (1 − 0.935 = 0.065) per each year spent in full-time education. The result is confirmed by a Mann–Whitney *U* test (*Z* = −10.399, *p* < 0.001).
- The probability of the answer ‘natural processes’ decreases with people perception that online or mobile communications can lead to misinformation. The probability decreases by 5.8% (1 − 0.942 = 0.058) per each more point scored in ‘Awareness of misinformation’. The result is confirmed by a Mann–Whitney *U* test (*Z* = −7.735, *p* < 0.001).
- The probability of the answer ‘natural processes’ is higher in men than in women.
- The probability of the answer ‘natural processes’ increases as people are more exposed to the news. The probability increases by 0.01% per minute of ‘News exposure’. The result is confirmed by a Mann–Whitney *U* test (*Z* = −2.313, *p* < 0.05). Data not normally distributed (Kolmogorov–Smirnov *p* < 0.05).

### The Full Model

In the *full model* the five options to answer the question ‘Do you think that climate change is caused by natural processes, human activity, or both?’, are present: 1—entirely by natural processes, 2—mainly by natural processes, 3—about equally by natural processes and human activity, 4— mainly by human activity, 5—entirely by human activity. As we now have five possible answers (categories), the model fit is a multinomial regression with a log link. The model was successfully fitted, and its parameterisation is presented in Table 4. The model uses the option ‘3—about equally by natural processes and human activity’, as the reference, while modelling the other options.

**Table 4:** Multinomial logit link regression parameterisation of the ‘full model’ fit with main effects and 2-way interactions

Climate change cause					95% Wald CI	
	Parameter	*β*	*p* value	Exp(*β*)	lower	upper
Entirely by natural processes	Intercept	−2.281	<0.01			
	Gender male	0.505	<0.001	1.656	1.326	2.069
	Awareness of misinformation	−0.167	<0.01	0.846	0.752	0.952
Mainly by natural processes	intercept	−1.575	<0.001			
	Gender male	0.304	<0.001	1.356	1.185	1.551
Mainly by human activity	Intercept	−1.446	<0.001			
	Awareness of misinformation	0.086	<0.001	1.090	1.046	1.137
	Years in education	0.083	<0.001	1.087	1.053	1.122
	Awareness of misinformation × Age	−0.001	<0.05	0.999	0.998	1.000
	Years in education × Age	−0.001	<0.01	0.999	0.998	1.000
Entirely by human activity	Intercept	−2.707	<0.001			
	Gender male	0.144	<0.05	1.154	1.033	1.290
	Years in education	0.097	<0.001	1.102	1.048	1.158
	Age	0.016	<0.05	1.016	1.002	1.030
	News exposure	−0.001	<0.05	0.999	0.999	1.000
	Years in education × Age	−0.002	<0.001	0.998	0.997	0.999

Likelihood ratio *χ*^2^ = 427, 28df, *p* < 0.001, AIC = 37,165. From the 18,060 individuals interviewed, 2025 individuals were excluded and 16,035 were included in the model

‘Awareness of misinformation’—‘How much online or mobile communications expose people to misinformation’, Years in education’—‘Years in full-time education completed’ ‘News exposure’—‘Time (minutes per day) spent watching, reading or listening to news about politics and current affairs’, ‘Climate change is caused equally by both natural processes and human activity’ is used as reference

**Fig. 2:**
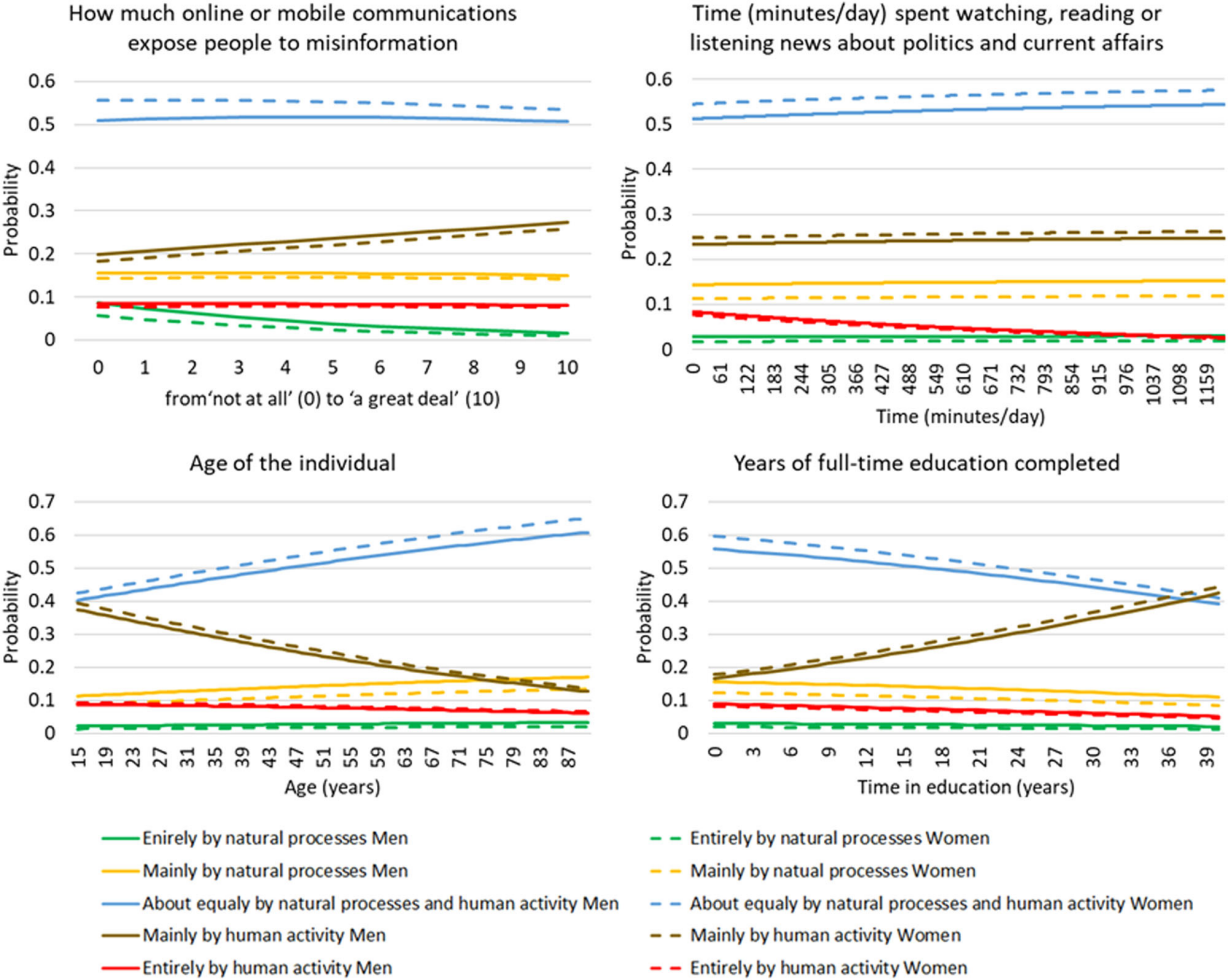
Representation of the multinomial logistic regression fit the model. Probabilities of answers to the question ‘Do you think that climate change is caused by natural processes, humanactivity, or both?’. Answers are a function of the covariable identified in the graph title. Due to the multidimensionality of the model, the graphs are drawn for one covariate while fixing the others in their mean values. The factor ‘Gender’ is represented in all the graphs

The model is also represented in Fig. 2; however, it must be noted that the multidimensionality of the model imposes drawing restrictions.

As the belief that online and mobile communications expose to misinformation increases the tendency to consider ‘mainly by human activity’, increases and ‘entirely by natural processes’ decrease as causes of CC.

People with a higher level of exposure to the news have a higher probability to answer that CC is caused ‘both by natural processes and human activity’ and a lower probability of answering ‘entirely by human activity’.

As individuals get older, the probability of answering ‘natural processes’ as the cause of CC increases. At the same time the answers ‘mainly by human activity’ and ‘entirely by human activity’ decrease their probability as age increases.

As the level of education increases, the probability of answering ‘mainly by human activities’ as the cause of CC also increases, while the answer ‘both by human activity and natural processes’ as causes declines with the level of education. More educated people also avoid the extreme answer ‘entirely by human activity’.

Women have higher probabilities to answer in favour of human activity causes, as both the answers ‘mainly by natural processes’ and ‘entirely by natural processes’ have lower probability as answers, when compared to men. Women have lower probabilities than men of answering ‘mainly by natural processes’ and ‘entirely by natural processes. The probability of answering ‘both by natural processes and human activity’ is also higher in women than men.

### The Negationists Model

In this model, the 114 individuals answering ‘I don’t believe climate change is happening’ are contrasted with those giving an answer for the causes of CC. The parameterisation of the model is shown in Table 5.

- The probability of denying CC increases if individuals spend less time watching, reading, or listening to news about politics and current affairs. The probability decreases by 0.3% (1 − 0.997 = 0.003) per minute less spent paying attention to the news.
- The probability of denying CC increases if individuals decrease their ‘trust in scientists’. The probability decreases by 11.1% (1 − 0.889 = 0.111) per each less score point given to ‘trust in scientists’.
- The probability of denying CC increases if individuals decrease their belief that ‘exposure to online/mobile communications exposes people to misinformation’. The probability decreases by 15.9% (1 − 0.841 = 0.159) per each less score point given to the belief.
- The probability of denying CC increases if individuals have fewer ‘years of full-time education completed’. The probability decreases by 18.7% (1 − 0.813 = 0.187) per year less spent in education.

**Table 5:** Logistic regression parameterisation of the ‘negationists model’

			95% Wald CI			95% Wald CI	
Parameter	*β*	Std error	lower	upper	Exp(*β*)	lower	upper
News exposure*	−0.003	0.0014	−0.006	−0.001	0.997	0.994	0.999
Trust in scientists**	−0.118	0.0303	−0.178	−0.059	0.889	0.837	0.943
Awareness of misinformation**	−0.173	0.0309	−0.233	−0.112	0.841	0.792	0.894
Years in education***	−0.208	0.0200	−0.247	−0.168	0.813	0.781	0.845

Likelihood ratio *χ*^2^ = 13480, 4df, *p* < 0.001, AIC = 1231; From the 18,060 individuals interviewed, 7452 are excluded and 10,608 are included in the model, from which 114 'don’t believe climate change is happening'

News exposure—‘Time (minutes per day) spent watching, reading or listening to news about politics and current affairs’, Years in education— ‘Years in full-time education completed’, Awareness of misinformation—‘How much online or mobile communications expose people to misinformation’; ‘Climate change is not happening’ is modelled, and ‘climate change is happening’ is the reference

*p < 0.05; **p < 0.01; ***p < 0.001

## Discussion

In the present study, we have explored the European citizens’ perceptions in relation to CC causes. In the first place, we aim to understand the factors shaping their perception in relation to the existence of CC, and secondly to understand the factors behind CC perception as a natural or anthropogenic process. We used a variety of explanatory variables to explain any variety observed in the results. The explanatory variables were divided into two groups, the demographic (age, gender, and level of education) and the society of information (exposure to news, internet, and mobile communications, and the level of trust in scientists). The results obtained show that only a small percentage (≈0.63%) of the population is in denial of climate change.

The model used to analyse people in denial shows that less educated people, with lower exposure to the news, with lower levels of trust in scientists, and with lower awareness of the probability of misinformation being passed in online and mobile communications, have a higher probability of being in denial. Age and gender were not significant in this model. Education has an obvious role in the development of critical thinking; therefore it is not a surprise that individuals with higher levels of education show higher levels of trust in scientists and therefore awareness of CC. A scientifically literate individual is also more capable of incorporating information and therefore consuming news more frequently. Also, more literate individuals, with higher levels of critical thinking are better equipped to spot and refuse misinformation passed online or by mobile communications (Huber et al. 2019). These results tally the results of previous research identifying the need for an educated society to tackle the CC challenge (Cordero et al. 2020).

The models fitted to the data considering the individuals believing in CC have identified several associations between the perception of CC of the European citizen and other IVs studied.

As individuals get older, the higher probability to think that CC is caused by natural rather than anthropogenic processes become evident. Younger people are more in agreement that CC has anthropogenic causes. Older individuals also have a higher probability of having lower levels of education as the levels of education have been growing consistently in European countries. Another possible reason for this effect is related to the levels of anxiety felt by younger generations in relation to CC. Clayton and Karazsia (2020) have studied this effect and had this conclusion, justifying it with the fact that younger individuals have a life to fulfil.

Being more educated and presenting higher levels of anxiety is also a result of exposure to scientific knowledge and higher critical thinking capacities, resulting in higher levels of trust in science and scientists. Our results show that more educated people have a higher probability of thinking that CC is caused mainly or entirely by human activities.

In our model, individuals believing CC is caused entirely by natural processes have a lower probability of trusting in scientists. Unlike Europe, in the US, numerous studies have been conducted on scepticism towards science and scientists regarding CC. In the United States trust in science and scientists is relatively high and about 84% of the adult population expressed a 'fair amount' or 'great deal' of confidence that scientists act in the best interests of the public, however, these figures vary with the level of education and more educated people show higher levels of trust (National Science Board 2022). In specific circumstances trust in science has become increasingly politicised (Bolsen et al. 2014; Goldfarb and Kriner 2017) with conservatist denying anthropogenic CC and democrats supporting it. In a study conducted in the Netherlands, (Rutjens and van der Lee 2020) found some association between CC scepticism and political conservatism, however at a lower scale than that observed in the US. Cologna and Siegrist (2020) in a metanalysis of studies made around the world, but mainly in Europe, found that the level of trust in science and scientists has a strong correlation with climate-friendly behaviours. In Western Europe, left-wing partisans tend to identify anthropogenesis as the main cause of CC. This divide is however not observed in Eastern Europe, in former communist countries (McCright et al. 2016).

News media are essential for individuals and society to understand, critically evaluate, and act on CC mitigation (Metag et al. 2017). Therefore the evaluation of the impact of the news in the public perception of CC is essential. However, studies show that in certain circumstances, the public may hesitate to take the messages conveyed by the news (Cheng and Gonzalez-Ramirez 2021). In Europe, media and high-level politics convey more accurate information (Muñoz and Sommer 2011). Our models show that Europeans reading, listening, or watching the news more frequently have a higher probability of believing that CC is caused by both natural processes and human activity. This tendency increases slightly with age, as younger individuals tend more for anthropogenic causes.

Sceptical individuals use online and mobile communication to convey misinformation, either by sharing or creating it (Van Der Linden 2022). Sceptical individuals doing so are more politically identified with conservationism and do not believe in anthropogenic causes of CC. Gerbina (2021) reported that CC fake news is among the most popular topics of science news fabricators. This author concluded from her study that social networks are dangerous powerful tools with significant propaganda potential that may be used in the manipulation of citizens’ minds. Social media platforms can often function as 'echo chambers' in which opinions on controversial issues can become more polarised (Grömping 2014). Our study shows that the probability of an individual thinking that CC is entirely and/or mainly caused by natural processes is lower in individuals thinking that exposure to mobile and online communications can convey misinformation. The tendency to consider that CC is caused mainly by human activity increases with the critical thinking that online or mobile communications expose people to misinformation. Therefore, disbelievers in CC anthropogenic causes are more exposed to fake online and mobile communication.

It has been previously reported that in Europe, concern and responsibility for climate change are more intense among women than men (Mata et al. 2023), which can be explained by the ‘conservative male’ effect or the higher frequency of male conservatist, as discussed by Jylhä et al. (2016). In this study, the probability of a European citizen perceiving CC causes as natural processes is higher in men than in women. Women have a higher probability of choosing human activity as the cause of CC.

In this study, we analyse the data from the ESS in four different models that represent different research perspectives. The way in which data is analysed can shape the results obtained and thus presenting different models is a characteristic that gives a more accurate picture of the issue than only showing an individual model. More interestingly, we show how while in some cases different variables are being selected by models, in many cases same variables are being selected and the direction of the effects is always coincident. Each model has potential uses to tackle particular problems, for example, an administration worker could be searching for research to make informed decisions to decrease climate change denialism in their region. As a limitation, we acknowledge the moment in which the 10th ESS was carried out may affect the results of the study. The survey took place during the COVID-19 pandemic in 2022, which could potentially modulate the degree of concern about CC and the perception of its threat. In a study to understand the worries behind reproductive decisions within climate crises across multiple age groups and gender, the ongoing pandemic was often seen as a much more tangible and important threat than climate change (Bodin and Björklund 2022).

## Conclusion

In general, the results from the 10th ESS show that the large majority of European citizens believe in climate change, however almost half of surveyed believe in both anthropogenic and natural causes of climate change. A significant proportion of the Europeans also consider a stronger contribution of anthropogenic causes with almost 45% considering human activity to be the entire or the main cause of climate change. We identified two key factors related to society of information that increase citizens’ belief in anthropogenic responsibility, namely the time spent reading news and the degree of awareness of online and mobile communication misinformation. In demographic terms, the typical individual that considers the human activity to be the main cause of CC is a young female with studies. These conditions also tend to come in hand with trust in science, more time spent watching or reading the news while having a developed sense of critical thinking on misinformation in current online and mobile communications.

These conclusions represent the analysis conducted with the average EU citizen in mind. However, we acknowledge the diverse cultural and political landscapes across various EU countries and regions. Further studies aimed at exploring the differences between EU countries and regions could serve as an excellent complement to the present study.

## Data Availability

The dataset analysed during the current study is available in the European Social Survey European Research Infrastructure Consortium repository, 10.18712/ess10e03_0.

## References

[CR1] Anderegg WRL, Prall JW, Harold J, Schneider SH (2010) Expert credibility in climate change. Proc Natl Acad Sci USA 107:12107–12109. 10.1073/pnas.1003187107 20566872 10.1073/pnas.1003187107 PMC2901439

[CR2] Bindoff NL, Stott PAA, AchutaRao, KM, Allen MRR, Gillett N, Gutzler D, Hansingo K, Hegerl G, Hu Y, Jain S (2014). Detection and attribution of climate change: from global to regional. In: Stocker TF, Qin D, Plattner G-K, Tignor M, Allen SK, Doschung J, Nauels A, Xia Y, Bex V, Midgley PM (eds.), Climate change 2013: The physical science basis. contribution of Working Group I to the fifth assessment report of the intergovernmental panel on climate change. Cambridge University Press, pp 867–952. 10.1017/CBO9781107415324.022

[CR3] Bodin M, Björklund J (2022) “Can I take responsibility for bringing a person to this world who will be part of the apocalypse!?”: ideological dilemmas and concerns for future well-being when bringing the climate crisis into reproductive decision-making. Soc Sci Med 302:114985. 10.1016/j.socscimed.2022.114985 35468522 10.1016/j.socscimed.2022.114985

[CR4] Bolsen T, Druckman JN, Cook FL (2014) The influence of partisan motivated reasoning on public opinion. Polit Behav 36:235–262. 10.1007/s11109-013-9238-0

[CR5] Buis A (2020) Milankovitch (orbital) cycles and their role in Earth’s climate. NASA Climate. https://climate.nasa.gov/news/2948/milankovitch-orbital-cycles-and-their-role-in-earths-climate/. Accessed 05 December 2023

[CR6] Busch T, Judick L (2021) Climate change—that is not real! a comparative analysis of climate-sceptic think tanks in the USA and Germany. Clim Change 164:18. 10.1007/s10584-021-02962-z

[CR7] Cheng H, Gonzalez-Ramirez J (2021) Trust and the media: perceptions of climate change news sources among US college students. Postdigit Sci Educ 3:910–933. 10.1007/s42438-020-00163-y

[CR8] Clayton S, Karazsia BT (2020) Development and validation of a measure of climate change anxiety. J Environ Psychol 69:101434. 10.1016/j.jenvp.2020.101434

[CR9] Collado S, Staats H, Sancho P (2019) Normative influences on adolescents’ self-reported pro-environmental behaviors: the role of parents and friends. Environ Behav 51:288–314. 10.1177/0013916517744591

[CR10] Collomb J-D (2018) A worthy heir: Donald Trump, the republican party and climate change. Revue LISA/LISA e-journal Littératures, Histoire des Idées, Images, Sociétés du Monde Anglophone–literature, history of ideas, images and societies of the English-speaking world, 16. 10.4000/lisa.9941

[CR11] Cologna V, Siegrist M (2020) The role of trust for climate change mitigation and adaptation behaviour: a meta-analysis. J Environ Psychol 69:101428. 10.1016/j.jenvp.2020.101428

[CR12] Cook J, Ellerton P, Kinkead D (2018) Deconstructing climate misinformation to identify reasoning errors. Environ Res Lett 13:024018. 10.1088/1748-9326/aaa49f

[CR13] Cordero EC, Centeno D, Todd AM (2020) The role of climate change education on individual lifetime carbon emissions. PLoS ONE 15:e0206266. 10.1371/2Fjournal.pone.0206266 32017773 10.1371/journal.pone.0206266 PMC6999882

[CR14] Dunlap RE, McCright AM, Yarosh JH (2016) The political divide on climate change: Partisan polarization widens in the U.S. Environ Sci Policy Sustain Dev 58:4–23. 10.1080/00139157.2016.1208995

[CR15] ESS ERIC (2022a) European Social Survey 10 - integrated file, edition 2.0. 10.18712/ess10e03_0

[CR16] ESS ERIC (2022b) ESS10 Data documentation. https://www.europeansocialsurvey.org/data/ Accessed 01 March 2023

[CR17] Farrell J (2016) Corporate funding and ideological polarization about climate change. Proc Natl Acad Sci 113:92–97. 10.1073/pnas.1509433112 26598653 10.1073/pnas.1509433112 PMC4711825

[CR18] Farrell J, McConnell K, Brulle R (2019) Evidence-based strategies to combat scientific misinformation. Nat Clim Change 9:191–195. 10.1038/s41558-018-0368-6

[CR19] Fernandez M, Alani H (2018) Online misinformation: challenges and future directions. Companion proceedings of the web conference, 595–602, 10.1145/3184558.3188730

[CR20] Gerbina TV (2021) Science disinformation: on the problem of fake news. Sci Tech Inf Process 48:290–298. 10.3103/S0147688221040092

[CR21] Goldfarb JL, Kriner DL (2017) Building public support for science spending: misinformation, motivated reasoning, and the power of corrections. Sci Commun 39:77–100. 10.1177/1075547016688325

[CR22] Grasso M (2019) Oily politics: a critical assessment of the oil and gas industry’s contribution to climate change. Energy Res Soc Sci 50:106–115. 10.1016/j.erss.2018.11.017

[CR23] Grömping M (2014) Echo chambers’: Partisan Facebook groups during the 2014 Thai election. Asia Pac Media Educ 24:39–59. 10.1177/1326365X14539185

[CR24] Gschnaller S (2020) The albedo loss from the melting of the Greenland ice sheet and the social cost of carbon. Clim Change 163:2201–2231. 10.1007/s10584-020-02936-7

[CR25] Hansen JE, Sato M, Simons L et al. (2023) Global warming in the pipeline. Oxf Open Clim Change 3:kgad008. 10.1093/oxfclm/kgad008

[CR26] Hegerl GC, Brönnimann S, Cowan T, Friedman AR, Hawkins E, Iles C, Müller W, Schurer A, Undorf S (2019) Causes of climate change over the historical record. Environ Res Lett 14:123006. 10.1088/1748-9326/ab4557

[CR27] Hornsey MJ, Harris EA, Bain PG, Fielding KS (2016) Meta-analyses of the determinants and outcomes of belief in climate change. Nat Clim Change 6:622–626. 10.1038/nclimate2943

[CR28] Hornsey MJ, Lewandowsky S (2022) A toolkit for understanding and addressing climate scepticism. Nat Hum Behav 6:1454–1464. 10.1038/s41562-022-01463-y 36385174 10.1038/s41562-022-01463-y PMC7615336

[CR29] Höttecke D, Allchin D (2020) Reconceptualizing nature‐of‐science education in the age of social media. Sci Educ 104:641–666. 10.1002/sce.21575

[CR30] Huber B, Barnidge M, Gil de Zúñiga H, Liu J (2019) Fostering public trust in science: the role of social media. Public Underst Sci 28:759–777. 10.1177/0963662519869097 31524092 10.1177/0963662519869097

[CR31] IPCC (2023) Climate change 2023 synthesis report, sections 1–4. In: Lee H, Romero J (eds.) Climate change 2023: synthesis report. contribution of working groups I, II and III to the sixth assessment report of the intergovernmental panel on climate change. IPCC, Geneva, Switzerland, 35–115. 10.59327/IPCC/AR6-9789291691647

[CR32] Jylhä KM, Cantal C, Akrami N, Milfont TL (2016) Denial of anthropogenic climate change: social dominance orientation helps explain the conservative male effect in Brazil and Sweden. Pers Individ Differ 98:184–187. 10.1016/j.paid.2016.04.020

[CR33] Knoblauch C, Beer C, Liebner S et al. (2018) Methane production as key to the greenhouse gas budget of thawing permafrost. Nat Clim Change 8:309–312. 10.1038/s41558-018-0095-z

[CR34] Kock H (2019) Best practices for attributing climate change to extreme weather events in media. Dissertation, University of Oregon, Eugene, Oregon, USA

[CR35] Lacey J, Howden M, Cvitanovic C, Colvin RM (2018) Understanding and managing trust at the climate science–policy interface. Nat Clim Change 8:22–28. 10.1038/s41558-017-0010-z

[CR36] Leal Filho W, Sima M, Sharifi A, Luetz JM, Salvia AL, Mifsud M, Olooto FM, Djekic I, Anholon R, Rampasso I, Kwabena Donkor F, Dinis MAP, Klavins M, Finnveden G, Chari MM, Molthan-Hill P, Mifsud A, Sen SK, Lokupitiya E (2021) Handling climate change education at universities: an overview. Environ Sci Eur 33:109. 10.1186/s12302-021-00552-5 34603904 10.1186/s12302-021-00552-5 PMC8475314

[CR37] Leggett JA (2020) The United Nations Framework Convention on climate change, the Kyoto Protocol, and the Paris Agreement: a summary. UNFCC, New York, USA, p 2

[CR38] Leiserowitz A, Maibach E, Rosenthal S, Kotcher J, Goddard E, Carman J, Verner M, Ballew M, Marlon J, Lee S, Myers T, Goldberg M, Badullovich N, Thier K (2023) Global warming’s six Americas, fall 2023. Yale University and George Mason University. Yale Program on Climate Change Communication, New Haven, CT, https://climatecommunication.yale.edu/publications/global-warmings-six-americas-fall-2023/. Accessed 16 February 2024

[CR39] Van Der Linden S (2022) Misinformation: susceptibility, spread, and interventions to immunize the public. Nat Med 28:460–467. 10.1038/s41591-022-01713-6 35273402 10.1038/s41591-022-01713-6

[CR40] Van der Linden S, Leiserowitz A, Rosenthal S, Maibach E (2017) Inoculating the public against misinformation about climate change. Glob Chall 1:1600008. 10.1002/gch2.201600008 31565263 10.1002/gch2.201600008 PMC6607159

[CR41] Machete P, Turpin M (2020) The use of critical thinking to identify fake news: a systematic literature review. In: Hattingh M, Matthee M, Smuts H, Pappas I, Dwivedi YK, Mäntymäki M (eds.), Responsible design, implementation and use of information and communication technology. Springer International Publishing, pp. 235–246. 10.1007/978-3-030-45002-1_20

[CR42] Mahalik MK, Mallick H, Padhan H (2021) Do educational levels influence the environmental quality? The role of renewable and non-renewable energy demand in selected BRICS countries with a new policy perspective. Renew Energy 164:419–432. 10.1016/j.renene.2020.09.090

[CR43] Mata F, Jesus MS, Cano-Díaz C, Dos-Santos M (2023) European citizens’ worries and self-responsibility towards climate change. Sustainability 15:6862. 10.3390/su15086862

[CR44] McCright AM, Dunlap RE, Marquart-Pyatt ST (2016) Political ideology and views about climate change in the European Union. Environ Polit 25:338–358. 10.1080/09644016.2015.1090371

[CR45] McKie RE (2019) Climate change counter movement neutralization techniques: a typology to examine the climate change counter movement. Socio Inq 89:288–316. 10.1111/soin.12246

[CR46] Metag J, Füchslin T, Schäfer MS (2017) Global warming’s five Germanys: a typology of Germans’ views on climate change and patterns of media use and information. Public Underst Sci 26:434–451. 10.1177/0963662515592558 26142148 10.1177/0963662515592558

[CR47] Muñoz M, Sommer B (2011) Perceptions of climate change: the role of art and the media. Boston. https://80000h.org/problem-profiles/climatechange/?utm_source=google&utm_medium=cpc&utm_campaign=80KMARGrantsClimateChangeBroad&utm_content=63198433170&utm_term=effects%20of%20climate%20change&gclid=Cj0KCQjwyLGjBhDKARIsAFRNgW_u7i3CSYhM9R078YZJJMf23ybHLe5j6Sj8k6ZCcYSFv6izo39qniQaAv5pEALw_wcB. Accessed 13 March 2023

[CR48] National Science Board (2022). The state of US science & engineering. Alexandria. https://ncses.nsf.gov/pubs/nsb20221. Accessed 13 March 2023

[CR49] Nunes LJR, Meireles CIR, Pinto Gomes CJ, Almeida Ribeiro NMC (2020) Forest contribution to climate change mitigation: Management oriented to carbon capture and storage. Climate 8:21. 10.3390/cli8020021

[CR50] Ojala M, Bengtsson H (2019) Young people’s coping strategies concerning climate change: relations to perceived communication with parents and friends and proenvironmental behavior. Environ Behav 51:907–935. 10.1177/0013916518763894

[CR51] O’Riordan T, Jäger J (2019) Beyond climate change science and politics. In: The Politics of climate change. Routledge, pp 346–360

[CR52] Painter J, Ashe T (2012) Cross-national comparison of the presence of climate scepticism in the print media in six countries, 2007–10. Environ Res Lett 7:044005. 10.1088/1748-9326/7/4/044005

[CR53] Parry M L, Canziani O, Palutikof J, Van der Linden P, Hanson C (2007). Climate change 2007-impacts, adaptation and vulnerability: working group II contribution to the fourth assessment report of the IPCC, vol 4. Cambridge University Press, New York, New York, USA

[CR54] Pepermans Y, Maeseele P (2018) Democratic debate and mediated discourses on climate change: from consensus to de/politicization. In: Media research on climate change. Routledge, New York, New York, USA, pp 88–104

[CR55] Rutjens BT, van der Lee R (2020) Spiritual skepticism? Heterogeneous science skepticism in the Netherlands. Public Underst Sci 29:335–352. 10.1177/0963662520908534 32126894 10.1177/0963662520908534 PMC7323769

[CR56] Shutaleva A (2023) Ecological culture and critical thinking: building of a sustainable future. Sustainability 15:13492. 10.3390/su151813492

[CR57] Stern DI, Kaufmann RK (2014) Anthropogenic and natural causes of climate change. Clim Change 122:257–269. 10.1007/s10584-013-1007-x

[CR58] Sundar S, Mishra AK, Shukla JB (2021) Effects of mitigation options on the control of methane emissions caused by rice paddies and livestock populations to reduce global warming: a modeling study and comparison with environmental data. J Environ Inf 38:106–115. 10.3808/jei.202000447

[CR59] Supran G, Oreskes N (2017) Assessing ExxonMobil’s climate change communications (1977–2014). Environ Res Lett 12:084019. 10.1088/1748-9326/aa815f

[CR60] Taimur S, Sattar H (2019). Education for sustainable development and critical thinking competency. In: Leal Filho W, Azul A, Brandli L, Özuyar P, Wall T (eds.) Quality education. Encyclopedia of the UN sustainable development goals. Springer. 10.1007/978-3-319-69902-8_64-1

[CR62] Tolppanen S, Claudelin A, Kang J (2021) Pre-service teachers’ knowledge and perceptions of the impact of mitigative climate actions and their willingness to act. Res Sci Educ 51:1629–1649. 10.1007/s11165-020-09921-1

[CR63] Whitmarsh L, Capstick S (2018) Perceptions of climate change. In: Psychology and climate change. Elsevier Academic Press, pp 13–33. 10.1016/B978-0-12-813130-5.00002-3

[CR64] Wong-Parodi G, Feygina I (2020) Understanding and countering the motivated roots of climate change denial. Curr Opin Environ Sustain 42:60–64. 10.1016/j.cosust.2019.11.008

[CR65] Zhang X, Ghorbani AA (2020) An overview of online fake news: characterization, detection, and discussion. Inf Process Manag 57:102025. 10.1016/j.ipm.2019.03.004

